# Cortical Merging in S1 as a Substrate for Tactile Input Grouping

**DOI:** 10.1523/ENEURO.0342-17.2017

**Published:** 2018-01-16

**Authors:** Julien Corbo, Yoh’I Zennou-Azogui, Christian Xerri, Nicolas Catz

**Affiliations:** Aix Marseille Univ, CNRS, LNIA UMR 7260, FR3C, Marseille, France

**Keywords:** Cortical processing, electrophysiology, optical imaging, S1, somatosensory, tactile

## Abstract

Perception is a reconstruction process guided by rules based on knowledge about the world. Little is known about the neural implementation of the rules of object formation in the tactile sensory system. When two close tactile stimuli are delivered simultaneously on the skin, subjects feel a unique sensation, spatially centered between the two stimuli. Voltage-sensitive dye imaging (VSDi) and electrophysiological recordings [local field potentials (LFPs) and single units] were used to extract the cortical representation of two-point tactile stimuli in the primary somatosensory cortex of anesthetized Long-Evans rats. Although layer 4 LFP responses to brief costimulation of the distal region of two digits resembled the sum of individual responses, approximately one-third of single units demonstrated merging-compatible changes. In contrast to previous intrinsic optical imaging studies, VSD activations reflecting layer 2/3 activity were centered between the representations of the digits stimulated alone. This merging was found for every tested distance between the stimulated digits. We discuss this laminar difference as evidence that merging occurs through a buildup stream and depends on the superposition of inputs, which increases with successive stages of sensory processing. These findings show that layers 2/3 are involved in the grouping of sensory inputs. This process that could be inscribed in the cortical computing routine and network organization is likely to promote object formation and implement perception rules.

## Significance Statement

When two close tactile stimuli are delivered simultaneously on the skin, subjects feel a unique sensation, spatially centered between the two stimuli. To understand the mechanism underlying this sensory merging, we investigated the S1 cortical representation of coincidental two-point stimuli. We demonstrated the phenomenon of cortical merging by which two distinct sensory inputs are represented by a unique and centered cortical activation. Our results, obtained with technical approaches capturing different stages of cortical processing, suggest that this merging is buildup as the sensory peripheral information travels along the S1 cortical network. By performing such merging, a fundamental role of S1 could be the grouping of distinct sensory stimuli into relevant perceptual objects.

## Introduction

Perception can be described as an inferential process, by which knowledge about what is to be perceived shapes the processing of sensory inputs (Gregory, 1997). It has been proposed that the rules guiding this perceptual construction are inscribed in the computational routine of the neural networks that process sensory inputs (Frégnac and Bathellier, 2015). A major challenge in the study of sensory systems is to understand these rules and shed light on their implementation. In this framework, illusions may result from erroneous inferences about the input, representing a situation where the rules become visible. As first described by von Békésy (1958, 1959), tactile funneling perception occurs when brief and simultaneous stimulation at multiple points aligned on the skin evokes a single sensation located at the center of the stimulus pattern. This centered sensation is more intense than a single stimulation at the central site, as if inputs from the edges are funneled into the center and increase the magnitude of the sensation (Gardner and Spencer, 1972a). Moreover, with two-point skin stimulation, a funneled sensation can be elicited at a central nonstimulated location (Sherrick, 1964; Chen et al., 2003).

Funneling perception reveals that the brain represents an array of simultaneous and close points as a single centered one, as if a continuous object has generated this pattern on the skin. How does this inference take place in the neural computation of tactile inputs? Gardner and Spencer (1972a) recorded mechanoreceptive afferent fibers and dismissed the involvement of peripheral mechanisms in this sensory funneling. The same authors (Gardner and Spencer, 1972b) examined primary somatosensory cortex (S1) single-unit responses to tactile stimuli presented simultaneously at three points on the skin. The neuronal population exhibited a broad excitation profile with a unimodal contour, instead of a trimodal one. These findings, however, did not account for the possibility that sensory funneling was induced without a central stimulation.

A more recent study using intrinsic signal optical imaging (ISOi) in area 3b in monkeys revealed that stimulation of two adjacent digits led to reduced activation at the sites of these digital topographic representations and to a centrally located activation that partially overlapped the cortical sectors activated by single-digit stimulation. However, this merging process was not observed for nonadjacent digit stimulation (Chen et al., 2003).

The rodent barrel cortex, whose columnar organization is precisely resolved, has been extensively investigated as a fruitful model of tactile cortical processing. Surprisingly, findings about the effects of simultaneous stimulation of adjacent whiskers on the response of barrel cortex neurons remain controversial. Some studies reported no change with respect to individual whisker stimulation (Goldreich et al., 1998; Shimegi et al., 1999). Other studies described a supralinear summation after three-whisker stimulation (Ghazanfar and Nicolelis, 1997) or a sublinear summation when four whiskers, but not two or three, were stimulated (Mirabella et al., 2001). These studies focused on the response magnitude and overlooked the spatial distribution of evoked neuronal activity. Chen-Bee et al. (2012) showed that four- and 24-whisker stimulations evoke cortical responses that are centered in a single location. This merging, explained by a simple linear additive interaction between inputs from the different whiskers, has been shown to result from cortical processing, as it is not passed on by subcortical structures. Based on these studies, one can propose the hypothesis of a built-in cortical merging of cutaneous inputs, which is transmitted through intracortical circuitries. Given that the temporal resolution of ISOi is constrained by neurovascular coupling, and that the description of S1 unit tuning curves and response characteristics of neuronal populations are lacking, the “built-in hypothesis” of cortical merging requires further substantiation.

To the best of our knowledge, the study by Chen-Bee et al. (2012) remains the only one investigating the merging process in the barrel cortex. The forepaw map, which is less discontinuous than the whiskers representation, may be better suited to elucidate the mechanisms of merging underpinning the funneling perception. To gain insights into the merging of two-point stimuli and its spatial limitation, we combined electrophysiological unit and local field potential (LFP) recordings with voltage-sensitive dye (VSD) optical imaging in the forepaw area of S1 while stimulating pairs of digits. Electrophysiological recordings were performed in the thalamocortical entry layer, 4 while VSD imaging primarily probed the synaptic activity in output layers 2/3 (Ferezou et al., 2006). Spatial diffusion of neuronal activity was analyzed from VSD and LFP data. A decoding procedure extracted spatial information from a neuronal population. Our findings sustain the view that cortical merging builds up as inputs pass through the cortical network laminar architecture.

## Materials and Methods

### Animal preparation

Principles of laboratory animal care were respected, and experiments were conducted in accordance with Directive 2010/63/EU of the European Parliament and of the Council of 22 September 2010 on the protection of animals used for scientific purposes. Every step of this experiment from design to implementation was made in accordance with a local ethics committee. Sixteen (8 for electrophysiological recordings, 8 for optical imaging) adult Long-Evans male rats weighing 350–500 g were used for acute recording. Anesthesia was induced with intramuscular medetomidine (0.25 mg/kg, Domitor, Orion Pharma) and ketamine (25 mg/kg, Virbac) injection. The level of anesthesia was monitored by testing the hind paw reflex and was maintained by injecting half of the original dose. Animal temperature was measured with an anal probe and regulated with a heated blanket. Once the animal’s head was fixed in a stereotaxic apparatus, an incision was made from bregma to lambda. Connective tissues and masseter muscle were removed, and craniotomy was performed to open a 5 × 5-mm window centered over the S1 in the right hemisphere. After the experiment, the rat received a lethal dose of embutramide (T61).

### Stimulation

The hairy side of the rat left forepaw was glued on a plate placed in a nearly vertical plane, perpendicularly to the table supporting the animal, and formed a natural angle with the forearm. The tips of shafts of custom-made electromagnetic stimulators were placed on the glabrous side of each of four fingertips with the help of a binocular microscope. The probes contacting the skin had a 1-mm^2^ surface area. These devices were individually controlled via a custom-made Matlab (RRID:SCR_001622) program via a PCI acquisition board (National Instruments). Stimulation consisted of ∼200–300-µm-square-wave pulse indentation and lasted 20 ms. The stimulation of single digits (D2, D3, D4, and D5) was randomized with the costimulation of two digits (D2D3, D2D4, and D2D5)

### Electrophysiology

Neural activity was acquired extracellularly using a 16-channel tungsten microelectrode array (MEA, Alpha-Omega). A reference and a ground electrode are located near electrode 1 and 8, respectively, reducing the uncertainty about the source location of the recorded LFP signal (Kajikawa and Schroeder, 2011). Two neighboring electrodes were separated by 250 µm. The 2 rows of 8 electrodes were also separated by 250 µm. The signal was amplified, filtered, and digitized with a commercially available neurophysiological system [multichannel acquisition processor (MAP), Plexon]. LFPs (1–200 Hz at 1 kHz) and spiking activity (400–5000 Hz at 22,000 kHz) were stored, single units were isolated, and the discrimination of waveforms was performed online, then refined offline using principal component analysis with commercially available software (Offline Sorter, Plexon).

### Analysis of unitary responses

For each unit, the mean evoked firing rate was extracted within a 40-ms time window starting 10 ms after the stimulation was delivered. The probability of response was also used, treating trials in which at least one action potential was fired within the 40-ms time window as positives, and those without any as negatives. A binomial test on these binary values was performed on each unit to determine its responsiveness to our stimuli, and possible differences between the responses to two different stimuli. A binomial distribution parameters estimate was made for each unit response to every stimulus, to determine a 95% confidence interval encompassing the real value of positive response probability (Matlab *binofit* function, using the Clopper–Pearson method). The decision was made by comparing the probability of a response to one stimulus with the interval from the binomial parameter estimate of another response. To assess responsiveness, the probability of responding to stimuli was compared to the confidence interval from the unit’s baseline. Each neuron was labeled with a preferred digit, which was the one that elicited its maximal probability of responding.

### LFP analysis

LFPs were used to assess whether the spatial distribution of activity after two-point stimulation was comparable to the activity after single-digit stimulation. Whenever we observed a clear somatotopic pattern of activity across the electrodes, encompassing the four stimulated digits, the files were kept for this analysis. For every electrode, the mean signal was smoothed using a Savinsky-Golay filter, derived, and rectified. The area under the curve (AUC) of these processed responses was used as a metric of the quantity of activation. For each block of stimulation, a linear regression was performed between the 8 data points from any single-digit stimulation and the ones from the two-digit stimulation. If the regression was significant (*F* test, *p* < 0.05), the correlation coefficient was considered as the *y* intercept. Two distributions of activity along the 8 electrodes that shared the same relative spatial distribution led to a significant correlation and a coefficient close to 1. The magnitude and sign of the *y* intercept carries information about amplitude variations independently of the relative spatial distribution.

### Decoding analysis

Decoding was performed using the *k* nearest neighbors (KNN) method from Matlab (*fitcknn* function). The procedure involved two steps: learning and testing. For *n* neurons, the learning step is to build an *n*-dimensional space to which all training trials are projected. Neural responses were simplified to binary values, according to the presence or absence of an action potential in a 40-ms time window starting 10 ms after the stimulus. Therefore, each trial was an *n*-length vector of 1s and 0s. For the testing step, in which predictions were made, test trials were projected in that *N*-dimensional space containing all training trials, and the most frequent label within the *k* nearest neighbors is predicted for the test trial.

The cortical surface assigned to the representation of each digit followed a D2–D5 gradient, with D2 being the widest. Data from S1 cartography work (Xerri and Zennou-Azogui, 2003) were used to quantify this gradient (D2’s surface = 1, D3 = 0.92, D4 = 0.76, D5= 0.69). Assuming that there was no difference in cell density across digit representations, these coefficients where used to pick neurons representing each digit accordingly. Therefore, for a total of *n* neurons = 118 for D2D5 decoding, and *n* neurons = 94 for D2D4 decoding, 35 D2 neurons were used, 32 for D3, 27 for D4, and 24 for D5.

Neurons were selected randomly among the whole population. Fifty subsets of 20 trials were randomly selected for each neuron. For each subset of 20 trials, all combinations of 19 + 1 trials were used to train and test the classifier. This whole procedure was repeated 100 times, leading to a total amount of 100 * 20 * 50 training and testing results for each training stimuli. Classification performance was assessed using the percentage of correct predictions represented in confusion matrices.

The Jaccard distance (Eqs. 1 and 2) was used to compute the distance between test point and training points, as it is particularly suited for vectors of binary values. *k* was set to 4, following the classic rule of thumb where *k* should be the square root of the number of observations used for the training (19):

(1) distance=1−J(A,B),

(2) $$\text{with}\;J\left(A,B\right)=\frac{\left|A\cap B\right|}{\left|A\cup B\right|}.$$

### Voltage-sensitive dye imaging

For VSDi experiments, anesthesia and surgical preparation to open both skull and dura over S1 were the same as described before. In addition, a tracheotomy was performed to maintain the rats under artificial respiration, and heart rate was measured. This way, imaging sequences could be synchronized to heartbeat and started during the ascending phase of respiration, improving the stability during acquisition and thus the signal/noise ratio. A hemostatic sponge soaked in a voltage-sensitive dye (RH 1691) and artificial cerebrospinal fluid solution was placed on the cortical surface. The sponge was resoaked in the dye after 1 h and left for another hour. After staining, the cortex was washed with isotonic physiologic serum, and a thin layer of 1% agar was applied to improve the stability and prevent the cortex from drying. Each trial consisted of 256 100 × 100-pixel images acquired at a 333-Hz rate with a high-speed CMOS based camera (MiCAM Ultima). Recordings blocks alternated trials with (stim trials) and without (blank trials) stimulation.

A Gaussian filter with a 1-pixel-wide SD was applied to smooth the data in both spatial dimensions of the frame. Evoked activation was retrieved by dividing the signal from consecutive stim and blank trials. The latencies of the evoked activation for each pixel were computed (Fig. 8*B*). We started by detecting, for each pixel, the frame for which the relative fluorescence changes (*DF*/*F*) value was significantly larger than baseline (*t* test, *p* < 0.05) and extracted the first-order linear relation by using the previously extracted *DF*/*F* and the *DF*/*F* corresponding to the frame T – 1: *F* = *at* + *b*, where *t* is the time (in milliseconds) and *F* is the *DF*/*F* value. The latency of the evoked activation was the extrapolated time for which *F* = 0. The latencies for each pixel were color-coded to generate the latency map, as exemplified Fig. 8*C*. The distribution of the 10,000 latencies was used to extract the *n* first activated pixels (Fig. 8*D*). The first activated pixels (FAP) area corresponded to the area containing this *n* pixels (Fig. 8*E*). Costimulation-evoked FAP were compared with that evoked by single stimulations, and with the sum of the concerned digits stimulated alone. These sums were obtained by adding the FAP area of the two digits, e.g., D2 and D3 for D2D3, and subtracting the overlap area between those digits.

## Results

### LFP

**Figure 1.**
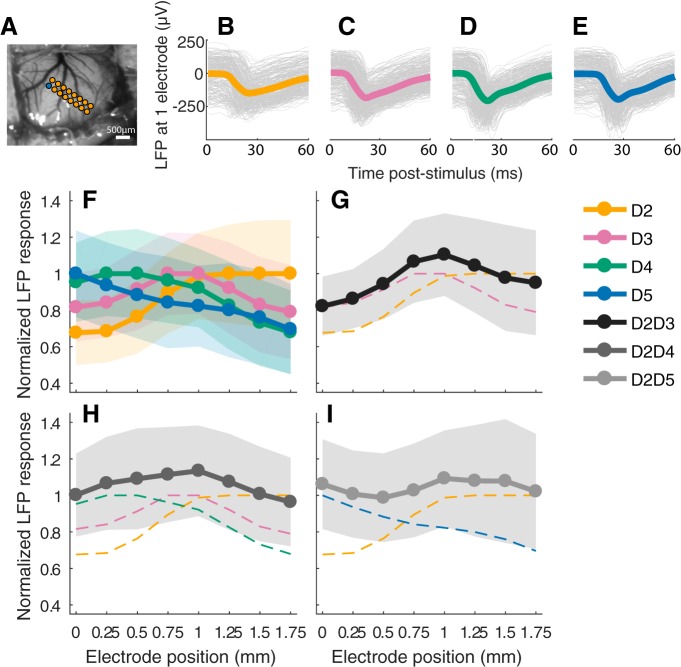
Example of S1 layer 4 LFPs recorded during single- and two-digit stimulations. ***A***, Location of the microelectrode array in S1. The blue dot represents the electrode at 0 mm. ***B–E***, Single trials (gray) and average (colored thick line) LFP responses for all single digit stimulations (D2–D5) recorded from the electrode marked with the blue dot. ***F***, Normalized mean area under the curve indicated as mean LFP response, according to recording electrode position (in millimeters). The colored transparent shapes indicate standard deviations. The spatial distribution of LFP responses was somatotopic, with each stimulation evoking a cortical activity profile gradually shifted along electrode position. ***G–I***, Funneled cortical pattern of activity elicited by digit costimulation (thick solid lines), superimposed on that generated by single stimulation of the same digits (dashed lines).

To compare the spatial distribution of cortical inputs elicited by one- or two-digit stimulation, normalized LFP responses were analyzed as a function of the relative electrode position along the digits representation (Fig. 1*A–E*). The expected somatotopic distribution of activity was confirmed (Fig. 1*F*). The LFPs at the most anterolateral electrodes showed a maximal response for D2. The greater LFP evoked by digit stimulation gradually moved along the electrode array from D2 to D3, D4, and finally D5 for the most posteromedial location. For D2 and D3 costimulation (D2D3), the peak of the activation pattern was located between the D2 and D3 evoked maximal responses (Fig. 1*G*). For D2 and D4 costimulation (D2D4), the same observation was made, and the maximal activity was located within the cortical zone of maximal responses to D3 stimulation (Fig. 1*H*). Such a centered activation pattern was not observed after the costimulation of the more distant D2 and D5, which elicited two response peaks, located where maximal responses were observed for D2 or D5 single stimulation respectively (Fig. 1*I*).

**Figure 2.**
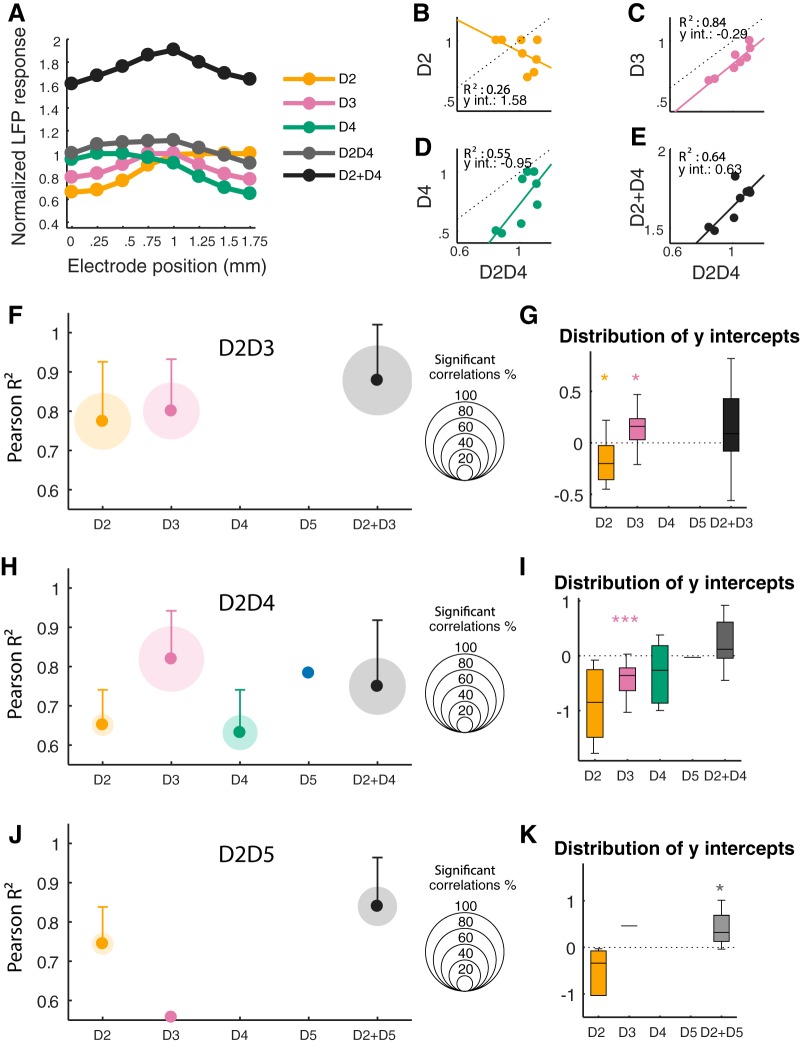
Regression analysis of LFP. ***A***, Example of mean LFP responses according to recording electrode position (see Fig. 1). Cortical pattern of activity evoked after D2, D3, and D4 single stimulation, D2D4 costimulation, and that obtained after the sum of D2 and D4 single-digit responses (D2 + D4). ***B–E***, Linear regression between D2D4 activity pattern and each of the patterns shown in ***A***. Note that the D2D4 costimulation-evoked activity pattern resembled that obtained after stimulation of the center digit, D3 (***C***). ***F***, ***H***, ***J***, Mean *R*^2^ (ordinate axis) and proportion of significant regressions (diameter of shaded discs; *F* test, *p* < 0.05) obtained in the whole neuronal population for each of the costimulation patterns (D2D3, D2D4, and D2D5). ***G***, ***I***, ***K***, *Y*-intercept distributions of the significant regressions between costimulation and single stimulation or addition activity patterns. A negative value indicates a greater activity for the costimulation (Wilcoxon test of the median versus 0; *p < 0.05; ***p < 0.001).

To quantify the similarities between the spatial distribution of different activation patterns, we performed linear regressions over eight AUC measures (area under curve of processed LFP response), corresponding to a line of recording sites for two stimulation conditions, whenever the line encompassed the representation of the four digits. Costimulation responses (D2D3, D2D4, and D2D5) were compared to single-digit responses (D2, D3, D4, and D5) and to the simple addition of the two single-digit responses, labeled D2 + D3, D2 + D4, and D2 + D5. Only the significant regressions were taken into account for this analysis (*F* test, *p* < 0.05). As exemplified for D2D4 costimulation (Fig. 2*A–E*), a close spatial relationship was found between D2D4 and either D3 (*R*^2^ = 0.84, *p* = 0.0015) or D2 + D4 (*R*^2^ = 0.64, *p* = 0.0175). The negative *y* intercepts for the regressions of D2D4 with D3 and D4 (–0.29 and –0.95, respectively) indicated that the activity elicited by the costimulation was greater than that evoked by the single stimulations. Nevertheless, the costimulation response appeared to be smaller than the addition of the D2- and D4-evoked responses (*y* intercept = 0.63). The correlation between D2D4 and D2 (*R*^2^ = 0.26, *p* = 0.192) or D4 (*R*^2^ = 0.55, *p* = 0.035) was weak.

At the population level, the proportion and mean *R*^2^ of significant regressions was taken as an index of similarity between the spatial distributions (Fig. 2*F–J*). Cortical input distribution after D2D3 costimulation was significantly correlated with D2 and D3 in 72% of the recordings (13/18, mean *R*^2^ = 0.77 for D2 and 0.80 for D3; Fig. 2*F*). This similarity was also revealed when considering the D2 + D3 addition. This comparison showed that 89% (16/18, mean *R*^2^ = 0.88) of D2 + D3 responses were significantly correlated with D2D3. Consistently, the proportion of significant regressions was paired with the mean *R*^2^ values. As expected, the D4 and D5 responses were in no case correlated with the D2D3 responses. Thus, the spatial distribution of cortical inputs for a D2D3 costimulation resembles both D2 and D3 related activity, as well as their addition.

The spatial distributions of D2D4 and D3 inputs were strongly correlated, with 75% (15/20) of significant regressions (mean *R*^2^ = 0.82; Fig. 2*H*). The negative median *y* intercept of these linear regressions indicates that the costimulation evoked a greater activation than D3 stimulation alone (–0.36, *p* = 1.221 × 10^−4^; Fig. 2*I*). The addition D2 + D4 also showed a similar spatial distribution of inputs and yielded 65% (13/20) of correlations (mean *R*^2^ = 0.75). D2 and D4, the effectively stimulated digits, yielded only 25% (5/20) and 40% (8/20) significant correlations with D2D4, respectively. As expected, the distribution of D5 stimulation–evoked LFPs was poorly correlated with that of D2D4 (1/20). Therefore, the spatial distribution of the layer 4 synaptic activity induced by D2D4 stimulation is close to that evoked by D3 and, to a lesser extent, by D2 + D4 stimulation.

For D2D5 costimulation, the only comparison yielding at least half of significant correlations was the D2 + D5 addition, with 9/18 and a mean *R*^2^ of 0.81 (Fig. 2*J*). The positive median *y* intercept for these regressions (0.32, *p* < 0.05) indicates a tendency for D2D5 costimulation to evoke a smaller activity than the addition of D2 and D5 responses (Fig. 2*K*). D2D5 spatial activity distribution was significantly correlated with that of D2 in 28% of the recordings (5/18) and D3 in only one case. No significant regression was found between D2D5 and D4 or D5 spatial patterns. Therefore, D2D5 spatial[[strike_start]] [[strike_end]]distribution of synaptic activity was similar to that resulting from the addition of D2 and D5, but was different from the ones elicited by the center stimulation D3 or D4.

#### Units

**Figure 3.**
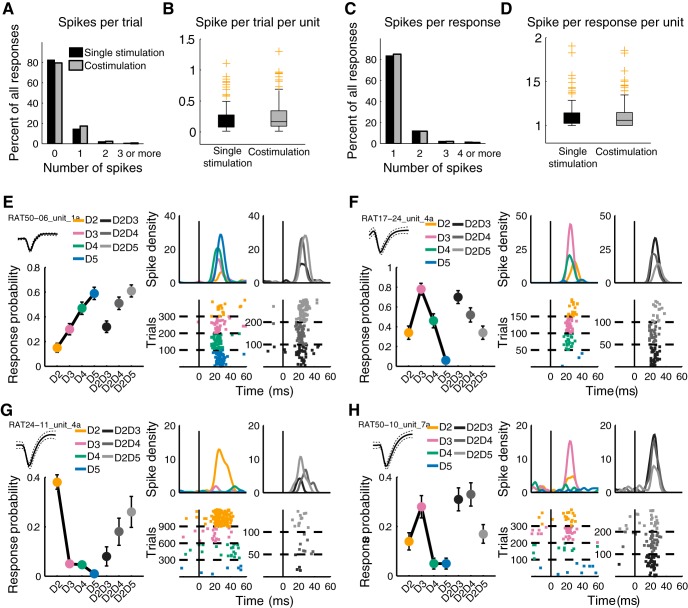
Single unit response properties and examples of recorded units. ***A***, Number of spikes per trial generated by single- and codigit stimulation for all neurons (*n* = 162) and all trials recorded. ***B***, Distribution of the number of spikes per trial and per unit. The median number of spikes is higher for the codigit than for the single-digit stimulation (Wilcoxon test, *p* = 0.000). ***C***, Number of spikes per response, i.e., for every trial in which at least one spike was generated. ***D***, Distributions of the mean number of spikes, per response per unit, for single-digit and codigit stimulation. The increased number of spikes per trial is not due to an increase in the number of spikes per response (Wilcoxon test, *p* = 0.54) but to an increased probability of response (***B***). ***E***, ***F***, Example of recorded units showing no additive or suppressive effect during costimulation. Left, mean wave form and neuron’s tuning curve represented as a mean response probability (± SD) for each single and codigit stimulation. Right, spike density function and raster plots for both single and combined stimulations (see color code matching digits in the diagrams). ***G***, Unit that shows a distance-dependent suppression after D2 stimulation with an adjacent or nonadjacent digit. ***H***, Unit responding to D2D3 and D2D4 costimulation as if D3, its preferred digit, were stimulated.

LFPs provide information about the distribution of synaptic inputs within layer 4. To gain insights into the spatial representation of costimulation, we investigated the discharge properties of single units in this layer. Of the 450 units recorded and sorted, 152 showed a significant response to at least one of the control stimuli, i.e., single-digit stimulation. The input increase suggested by our LFP data was paired with an increase in the response probability of units (Wilcoxon test, *p* = 0.0001; Fig. 3*A*,*B*). Before analyzing the spatial representation (digit location) embedded in the discharge of the recorded units, we needed to characterize their response properties. The large majority of units’ responses to any digit stimulation was a single spike (84%; Fig. 1*C*). Hence, the probability of responding was used in the subsequent analyses. Unit responses were considered as binary variables, and a binomial test was used to compare the responses to different stimuli. They were sorted according to their preference, i.e., the digit whose stimulation elicited the maximal probability of response.

In the recorded population, neurons displayed different types of modulation to digit costimulation compared with single digit stimulation. The units “preferring” the digits effectively stimulated, namely the “edges” of the stimulus pattern, could either decrease their probability of response when their preferred digit was stimulated with a second one (Fig. 3*G*) or fire as if they were stimulated alone (Fig. 3*E*). The neurons representing the center digits could either increase their probability of response to the edge digits (Fig. 3*H*) or display no change (Fig. 3*F*).

**Figure 4.**
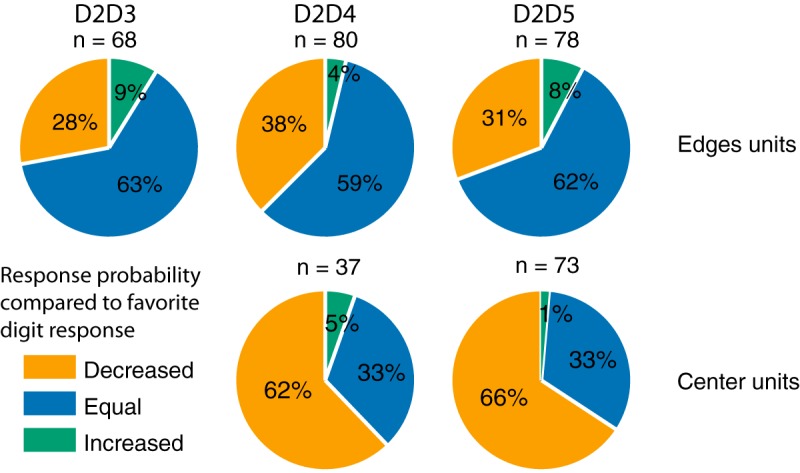
Population response modulation to costimulation. Proportion of units showing increased, decreased, or equal response probability to costimulation compared with that elicited by the stimulation of their preferred digit. Top row, edge units, i.e., with a maximal response probability to the stimulation of one of the costimulated digits. Bottom row, center units, i.e., with a maximal response probability to the center nonstimulated digit.

At the population level, most units representing the edges of the stimulation pattern did not respond differently than when they were activated by a single stimulation of their preferred digit (63% for D2D3, 59% for D2D4, 62% for D2D5; Fig. 4, upper row). The most remarkable effect for these units was a suppression in activity. When D2 and D3 were costimulated, the probability of response of 28% of neurons preferring D2 or D3 decreased. Consistently, 38% of the neurons preferring D2 or D4, and 31% of the neurons preferring D2 or D5, responded with a lower probably to their costimulation, respectively D2D4 and D2D5. Therefore, a substantial proportion of units representing the edges of the stimulation decreased their probability of responding, the larger effect being observed after a D2D4 costimulation. The units with their maximal response on the center digits also displayed such a variety of modulations. After a D2D4 costimulation, 62% of D3 preferring neurons responded less than they did for their preferred digit (Fig. 4, lower row). This result is not surprising, as D3 was not stimulated in that case. In contrast, the remaining 38% of neurons preferring D3 responded as if D3 were stimulated, or even more (Fig. 4, lower row).

Compared with the stimulation of D2 or D4 alone, 64% of these center neurons fired less than for D3, but for 37% of them the binomial test could not differentiate the responses to D3 and an adjacent digit. Therefore, the proportion of D3 neurons that fired as if D3 were stimulated could not be a consequence of the costimulation itself, but of the uncertainty of their tuning curve. To address this problem, we compared the identity of the D3 units that reported D2D4 as D3 with the units that could not differentiate D2 or D4 and D3. This comparison indicated that these units were not necessarily the same, as 8 of 14 that fired in a D3-like fashion could clearly discriminate adjacent digits around D3. For D2D5 costimulation, center neurons, preferring D3 or D4, yielded comparable results but with a slightly weaker effect of costimulation. 66% of them responded with a lower probability after D2D5 than after their favorite digit stimulation, D3 or D4, and the remaining 34% responded equally, or even more when their preferred digit was stimulated alone.

Even if such a classification of edge and center neurons does indicate a clear tendency for funneling, it does not inform us about the magnitude of the observed modulation of discharge probability. Could the decreasing edges neurons and increasing center neurons drive the whole population of neurons toward a centered, funneled pattern of activity?

#### Population analyses

**Figure 5.**
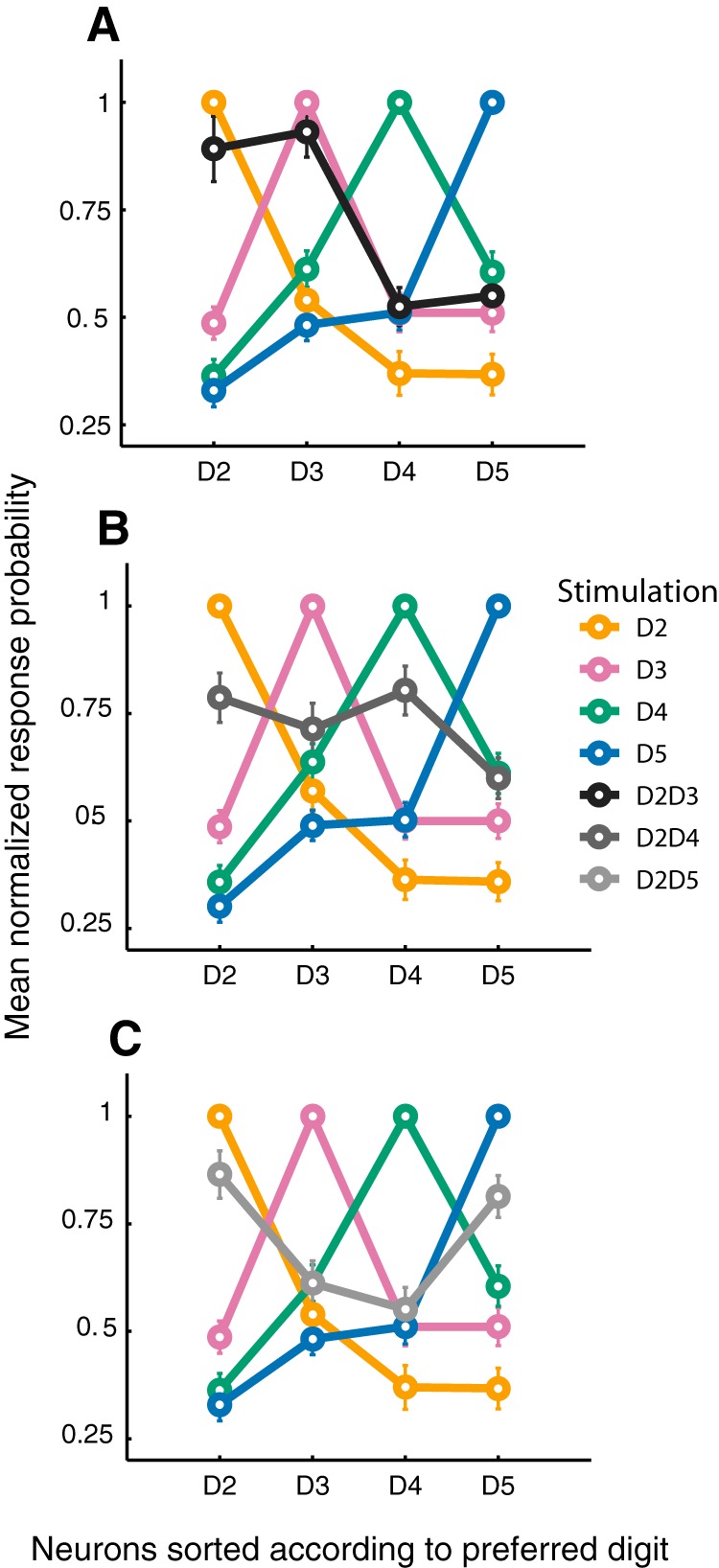
Cortical pattern reconstruction from the population responses. Mean normalized response probability (± standard error) to different stimulation conditions for neuronal populations sorted out according to the unit’s preferred digit, i.e., the digit whose stimulation elicited the highest probability of response. Population patterns for all neurons recorded after single-digit stimulation and D2D3 (***A***), D2D4 (***B***), or D2D5 (***C***) costimulation.

Given such idiosyncratic responses in layer 4 after costimulation of two adjacent (D2D3) or nonadjacent (D2D4 and D2D5) digits, could a neuronal population averaging reflect the buildup of cortical output and the downstream elaboration of a merged pattern, possibly leading to a merged perception? Sorting the neurons according to their preference allowed us to visualize the population average activity on a recreated cortical space, by reconstructing the somatotopic distribution of the units. The maximal normalized population response probability was reached for neurons preferring the stimulated digit. The response probability decreased as a function of distance from this peak (Fig. 5).

The population of neurons preferring D2 showed a similar, yet lower, probability of response to D2D3 than to D2 (D2D3, mean ± SEM 0.20 ± 0.02, versus D2, 0.23 ± 0.02; paired *t* test performed on nonnormalized data, *p* = 0.0494; Fig. 5*A*). The population of D3 neurons demonstrated the same tendency, but no significant difference was revealed (D3, 0.29 ± 0.04, versus D2D3, 0.27 ± 0.04; *p* = 0.14). Further, there was no significant difference between the firing probability for D2 and D3 neurons after the costimulation (0.20 ± 0.02 vs. 0.27 ± 0.03, *t* test, *p* = 0.089), creating a representational situation that corresponded to none of the single-digit stimulation. Indeed, the populations of D2 neurons and D3 neurons responded differently regardless of the stimulation, D2 or D3 (responses to D3, D3 population mean ± SEM, 0.29 ± 0.03, vs. D2 population, 0.12 ± 0.02, *t* test, *p* = 7.228 × 10^−5^; responses to D2, D2 population 0.23 ± 0.02 vs. D3 population, 0.17 ± 0.02; *p* = 4.478 × 10^−3^). As we chose to sort units according to their preferred digit, it was not possible for us to evaluate the behavior of units putatively at the frontier of D2 and D3 representation.

After D2 and D4 costimulation, neurons representing the edges (D2 and D4) fired with a lower probability, whereas center neurons increased their response compared to D2 and D4 single stimulation (Fig. 5*B*). D2 neurons fired with a lesser probability for D2D4 than for their favorite digit (D2, 0.26 ± 0.03; D2D4, 0.212 ± 0.026; paired *t* test, *p* = 0.001). The same modulation was reported for D4 population (D4, 0.27 ± 0.03; D2D4, 0.23 ± 0.03; *p* = 0.002). Regardless of such a decrease in firing probability, both D2 and D4 population responses were still clearly above their spiking probabilities for the stimulation of D3 (D2 population, response to D3, 0.14 ± 0.02; response to D2D4, 0.21 ± 0.02; paired *t* test, *p* = 5.07 × 10^−4^; D4 population, response to D3, 0.15 ± 0.02; response to D2D4, 0.23 ± 0.03; *p* = 9.76 × 10^−9^). D3 population discharged more for D2D4 than for D2 and D4 (pooled responses to D2 and D4, 0.20 ± 0.02 vs. D2D4, 0.25 ± 0.03; *p* = 0.0021). This higher response probability of D3 population to the D2D4 costimulation did not reach the probability yielded by D3 stimulation (D3, 0.31 ± 0.03; *p* = 1.72 × 10^−4^). After the D2D4 costimulation, the D2, D3, and D4 populations exhibited a similar probability of response.

**Figure 6.**
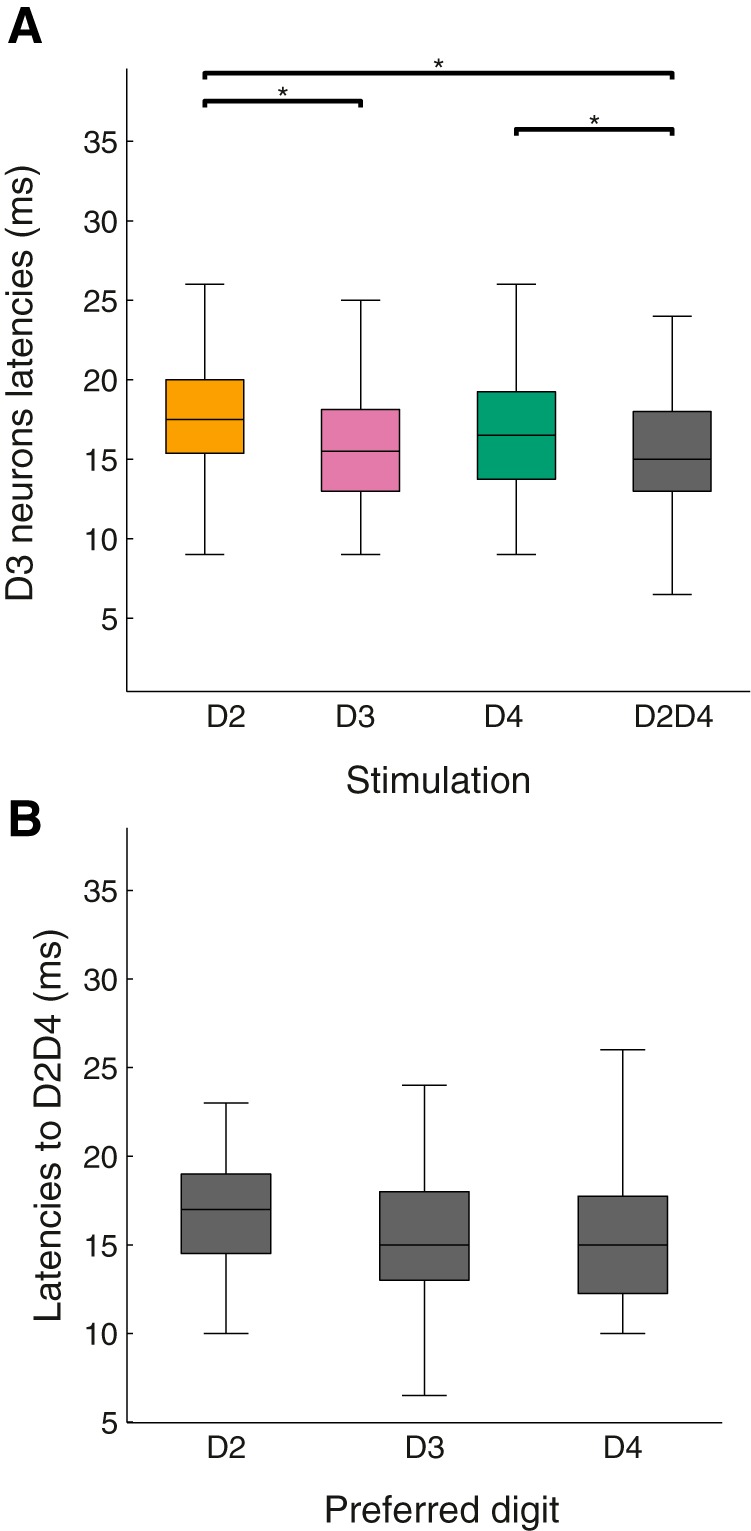
Unit response latency. ***A***, Distribution of the first spike latency after single-digit and D2D4 stimulations for D3-preferring neurons. Note that these neurons responded to D2D4 as fast as to D3. ***B***, Distributions of the first spike latency of the D2-, D3-, and D4-preferring neurons for D2D4 costimulation. There was no significant latency difference among these populations.

As the most efficient stimulation usually elicits a shorter first-spike latency (Armstrong-James et al., 1991; Moxon et al., 2008), we speculated whether the activity from the D3 population was temporally comparable to the one evoked by D2 and D4 alone, or to D3. It appeared that D3 neurons’ first-spike latencies to D2D4 stimulation (Fig. 6*A*) was shorter than those to D2 and D4 stimulation, and comparable to D3 (median for D2, 17.75 ms; D3, 15.5 ms; D4, 16.5 ms; D2D4, 15 ms; Wilcoxon signed rank test, D2D4 vs. D2, *p* = 0.0032; D2D4 vs. D4, *p* < 0.0125). D2 and D4 neurons did not show this latency facilitation for the costimulation (D2 and D2D4 median value, 17 ms; D4 and D2D4, 15 ms; Fig. 6*B*). When projected on the somatotopic cortical space, a decrease of the number of edge neurons responding to D2D4, coupled with an increase of active center neurons, led to a broader pattern of activation than the one elicited by single-digit stimulation alone. This broader population pattern was accompanied by a decrease in its sharpness, possibly leading to loss of sensory contrast.

After D2D5 costimulation, the population response resembled the predicted response to D2 and D5 single stimulation. The D2 population probability of firing in response to D2D5 stimulation was close to, yet significantly lower than, that elicited by D2 stimulation (D2D5, 0.20 ± 0.02 vs. D2, 0.23 ± 0.02; paired *t* test, *p* = 0.0453). Consistently, the D5 population probability of firing in response to D2D5 stimulation was lower than that of D5 alone (0.26 ± 0.03 vs. 0.30 ± 0.31; *p* = 0.0051). Thus, there was a slight decrease of response probability for the units representing the edges of the stimulation pattern. D3 neurons did not show such a significant decrease in spike probability compared to their D2 response (0.19 ± 0.03 vs. 0.17 ± 0.02; *p* = 0.164), which was also observed for D4 neurons compared to their D5 response (0.18 ± 0.03 vs. 0.15 ± 0.03; *p* = 0.079). The decrease found for the edge units was not accompanied by an increase in firing of the center units, as observed for D2D4 costimulation.

#### Decoding

**Figure 7.**
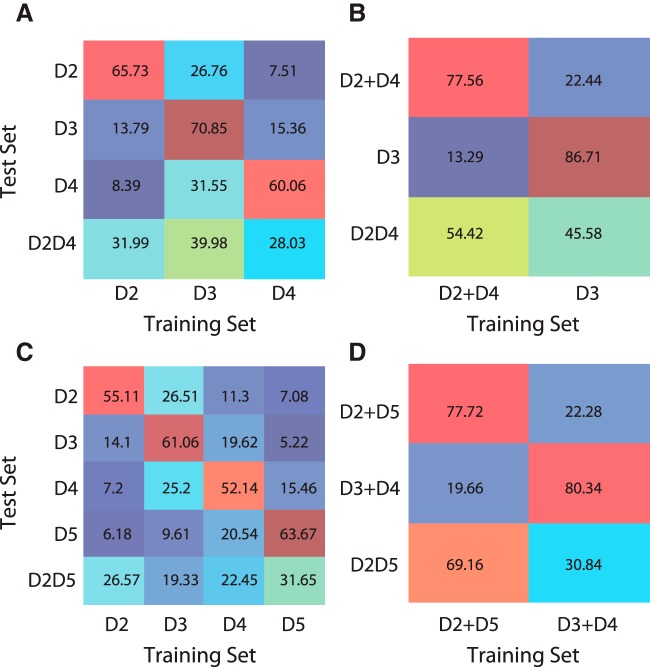
Decoding results. Confusion matrices obtained with a *k* nearest neighbors classifier (*k* = 4). ***A***, D2D4 population response classification. The classifier was trained with D2, D3, and D4 trials. Among the whole population, 94 neurons were chosen randomly, and 50 random subsets of 20 trials were used for each selected neuron. For every subset of 20 trials, all 20 different combinations of 19 + 1 were used to train and test the classifier. This procedure was repeated 100 times. The confusion matrices show the distribution of the 100,000 (100 * 50 *20) classifier choices. The most often chosen digit is D3, the center nonstimulated digit (39.98% > 33.33%, chance level). ***B***, Confusion matrix for a classifier trained with D3 and D2 + D4 trials. Neurons with their maximal response probability for D2 and D4 stimulation were grouped, as for trials where these digits were stimulated alone. This merging creates an artificial condition in which responses of D2 population for D2, and responses of D4 population for D4, are added. This condition allows us to verify whether the classifier would chose D3 over the simple addition of D2 and D4 responses. Decoding results indicate that addition of D2 + D4 was chosen more often. ***C***, D2D5 population response classification. The classifier was trained with D2, D3, D4, and D5 trials. ***D***, Confusion matrix for a classifier trained with D2 + D5 and D3 + D4, which indicated an advantage for the addition of edge digits’ responses.

We speculated whether the reported modulation of population response could lead to a mismatch between the stimulated digits and the spatial information embedded in the neuronal response. Could the center digits (e.g., D3) be read out of this population activity (e.g., when D2D4 is stimulated), and be represented in the next stages of sensory processing, resulting in percept-related responses? To address this question, we trained a *k* nearest neighbor (KNN) classifier to examine the extent to which the D2D4 and D2D5 costimulation single trials were in proximity to every other single digit stimulation. A classifier trained with three (D2, D3, and D4) digits was used to decode D2D4 trials (Fig. 7*A*,*B*), and one trained with four digits (D2, D3, D4, and D5), to decode D2D5 trials (Fig. 7*C*,*D*).

The classifier performed well on controls, recognizing each single stimulation above chance level (D2, 65.73%; D3, 70.85%; D4, 60.06%; Fig. 7*A*). For D2D4 trials, the decision made by the classifier was not as clear as for controls, although D2D4 trials were classified as D3 in 39.98% of the cases, the only one above the 33% chance level (D4, 28.03%; D2, 32%). Not only was the nonstimulated center digit chosen by the classifier, but it was also chosen with the greatest probability. Given that we forced the decision toward single stimulations, we wanted to test whether the classifier would choose the center digit D3 versus the edges D2 + D4 grouped in one pool. Consistently, the responses of D4 neurons to their preferred digit were labeled as if they were evoked by D2 stimulation. The population response to D2 was then similar to the addition of the single stimulations of both D2 and D4, without any interaction or modification due to a real costimulation. The KNN classifier was trained with this edge-response pool and with D3 responses. When fed with D2D4 trials, the pooled edges were chosen over D3 (54.42% vs. 45.58%).

As predicted from the population pattern of activity, D2D5 trials were not classified as center digits (with D3 and D4 19.33% and 22.45%, respectively). The classifier postdicted that D2 (26.57%) or D5 (31.65%) was stimulated, both being above the 25% chance level. As for D2D4, we tested whether the classifier would choose a D2 + D5 edges pool over a D3 + D4 center pool. In contrast to D2D4 trials, the classifier clearly chose the edges rather than the center (69.16% vs. 30.84%)

### Optical imaging

**Figure 8.**
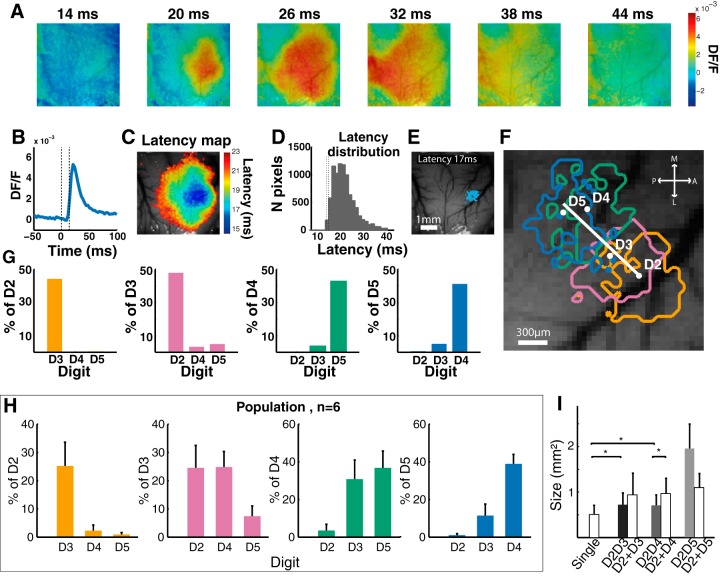
Voltage-sensitive dye imaging of cortical responses. ***A***, Example of cortical activation dynamics evoked by digit stimulation. ***B***, Example of mean *DF*/*F* over time for one pixel. ***C***, Latency map with cold (blue) colors representing the shortest latencies. ***D***, Latency distribution of all pixels of the image of activation shown in ***C***. ***E***, First activated pixels (FAP) for a single digit stimulation. ***F***, Example of FAP contours for all single digits, showing a somatotopic organization of the distal phalange of finger representations. ***G***, Percentage of overlap between FAP elicited by single-digit stimulations, for one animal. ***H***, Mean percentage of FAP overlaps for the eight animals included in the study. ***I***, Mean FAP areas for single stimulations, costimulations, and sums of single stimulations (paired Wilcoxon test, *p < 0.05).

We investigated the influence of the described firing pattern modifications to reveal whether they impacted the representation of the stimulations at the next stage of the columnar processing (i.e., in the superficial layers II and III), VSDi was used to directly extract the spatial region of cortical activity evoked by two-point costimulation. Single-digit stimulation evoked a focal activation for few milliseconds, which then spread over almost the entire imaging window (Fig. 8*A*). As these large propagations were found, analyzing the location of pixels for which the relative fluorescence changes (*DF*/*F*) reached a given threshold would lead to overlaps of digital representations. To circumscribe the cortical regions activated by digit costimulation within the somatotopic map, the topology of cortical activation had to be extracted before the single digit–related activations spread out over the adjacent regions. For this purpose, we computed the latency of *DF*/*F* above-threshold increase for each pixel (Fig. 8*B*) and built a latency map (Fig. 8*C*) revealing the location of the first activated pixels (FAP; Fig. 8*D*,*E*).

The centroids of the D2–D5 single stimulation FAP were distributed along the rostrocaudal axis, in keeping with well-known cortical somatotopy (Fig. 8*F*). Furthermore, the amount of overlap between adjacent digit representations decreased with the distance between the digits (Fig. 8*G*). For example, in the illustrated case, although 44.7% of the pixels within D2 representation were shared with the representation of D3, this percentage dropped to 0 when considering the overlap with D4 or D5 (Fig. 8*G*). At the population level (*n* = 8 animals), the representations of neighboring digits shared 20–40% of the cortical surface (*n* = 8, Fig. 8*H*). The sizes of D2, D3, D4, and D5 FAP surfaces were not significantly different from one another (Kruskal–Wallis test, *p* = 0.54). The pooled sizes for individual-digit FAP areas were smaller than those obtained after costimulations (mean ± SD, single digits, 0.51 ± 0.20 mm^2^; D2D3, 0.72 ± 0.26 mm^2^; Wilcoxon test, *p* = 0.031; D2D4, 0.71 ± 0.23 mm^2^; *p* = 0.031; D2D5, 1.96 ± 0.54). This size increase is consistent with the single unit data showing that costimulation evoked a greater cortical activity than single-digit stimulation. Compared with the sum of the FAP resulting from the single stimulations of the concerned digits, D2D3 FAP covered an area that tended to be smaller than D2 + D3, but not significantly (D2D3, 0.72 ± 0.26 mm^2^; D2 + D3, 0.94 ± 0.47 mm^2^; *p* = 0.093). D2D4 FAP area was smaller than D2 + D4 (0.97 ± 0.34; *p* = 0.031). D2D5 induced a dramatically larger size of activation, spanning across the whole distal finger pad representations. Accordingly, D2D5 FAP area was greater than D2 + D5 (1.1 ± 0.31, Fig. 8*I*).

**Figure 9.**
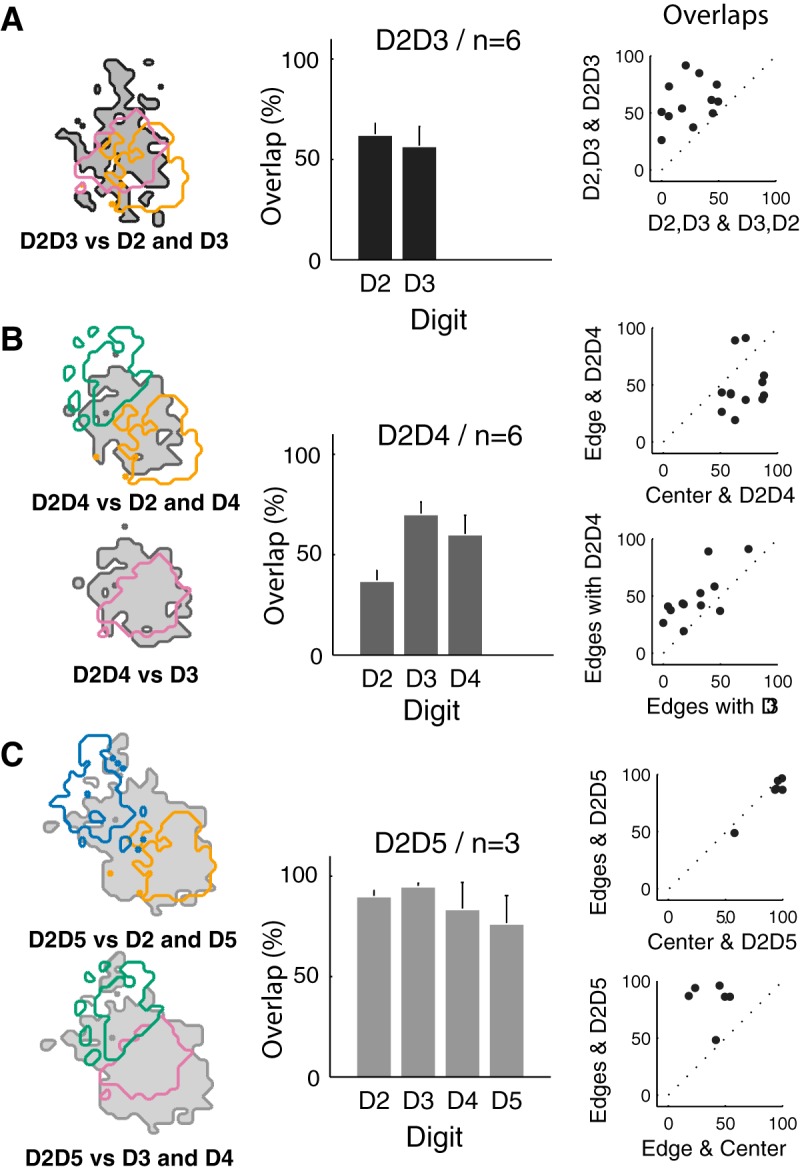
Comparison of the first activated pixel (FAP) areas in response to costimulation and single stimulation. ***A***, Left, example of FAP contours for D2D3 (gray), D2 (yellow), and D3 (pink) stimulations. Center, mean percentage of overlap between D2D3 and D2 and D3 FAP areas. Right, scatter plot of each animal overlap percentages between D2D3 and D2 or D3 versus overlaps between D2 and D3 or D3 and D2. ***B***, Left, examples of FAP contours for D2D4 (gray), D2 (yellow), and D4 (green) and for D2D4 (gray) and D3 (pink) stimulations, respectively, in the top and the bottom. Center, mean percentage of overlap between D2D4 and D2, D3 and D4 FAP areas. Right, scatter plot of individual FAP overlap percentages between D2D4 and edge digits (D2 and D4) versus D2D4 and D3 (top); between D2D4 and edge digits versus D3 and edge digits (bottom). ***C***, Left, examples of FAP contours for D2D5 (gray), D2 (yellow), and D5 (blue) and for D2D5 (gray) versus D3 (pink) and D4 (green) stimulations, respectively, in the top and the bottom. Center, mean percentage of overlap between D2D5 and D2, D3, D4, and D5 FAP areas. Right, scatter plot of individual overlaps percentages, between D2D5 and edge digits (D2 and D5) versus edge digits and center digits (D3 and D4; overlaps between adjacent digits; top); between center digits and edge digits versus D2D5 and center digits (bottom).

The early activation induced by two-digit stimulation indicated a cortical merging of inputs, for every pair of digits. The FAP after D2D3 costimulation were located centrally to the D2 and D3 representations (Fig. 9*A*). D2D3-evoked early activation enclosed 62.15 ± 5.75% of the D2 pixels, and 56.42 ± 9.95% of D3 pixels (Fig. 9*A*). The FAP area of the D2D3 costimulation was found to overlap more with the neighboring-digit FAP area than with that resulting from a single stimulation but did not encompass the whole D2 and D3 FAP areas (Fig. 9*A*, right column; ranked Wilcoxon test, *p* = 0.031).

Consistently, the D2D4 early activation area overlapped greatly with the central nonstimulated digit D3 FAP (Fig. 9B, left column). D2D4-evoked FAP area enclosed 70.04 ± 6.14% of pixels of D3 representation, but only 36.76 ± 5.47% and 59.95 ± 9.7% of D2 and D4 FAP, respectively. The FAP area of the D2D4 costimulation extended more over the edge digits FAP area than did the D3 single stimulation (Fig. 9*B*, right column; ranked Wilcoxon test, *p* = 0.006). However, the D2D4 FAP area only partially encompassed the D2 and D4 FAP areas. Moreover, D2D4 overlapped more over D3 than D2 or D4 areas. D2D5 costimulation evoked a single large activation that nearly encompassed all four digital representations. D2D5 FAP area enclosed 89.5 ± 3.12%, 94.64 ± 1.9%, 83.5 ± 13.35%, and 76.20 ± 14.11% of D2, D3, D4, and D5 FAP pixels, respectively (Fig. 9*C*).

**Figure 10.**
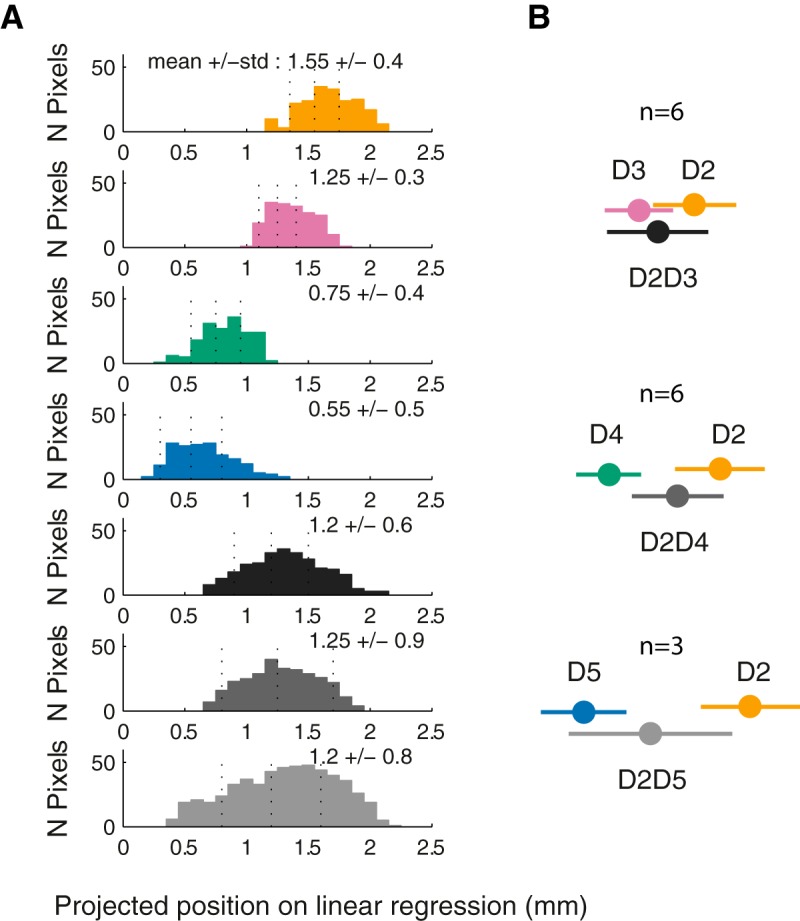
Location of costimulation-evoked first activated pixel (FAP) area along the somatotopy axis. ***A***, Example of distributions of FAP areas projected onto the somatotopy axis. The axis was determined by performing a linear regression onto the single-digit FAP areas centroids. ***B***, Single-digit and codigit stimulation–evoked centered activations. Points represent the mean of FAP distributions for the population, with bars indicating the mean and SD.

To obtain the precise location of the costimulation FAP along the somatotopic axis, a linear regression was performed over the four single-digit centroids for each animal. All FAP were then projected orthogonally onto this axis, allowing us to describe the distribution of FAP, and thus compare the location of activity induced by single- versus two-point stimulation (Fig. 10). In the example depicted in Fig. 10*A*, the digits’ representations were well differentiated. For the single-digit FAP areas (D2–D5), the colored distributions of the projected pixels onto the linear regression were located on different positions on the reconstructed axes. All pairs of costimulation evoked unimodally distributed activations. Their means were located centrally, between the stimulated digits (Fig. 10*B*). This direct spatial measure supports the overlap analyses and confirms that costimulation of adjacent and non-adjacent digits evoked unique and centered activations.

## Discussion

### Cortical representation of two-point stimuli

To characterize the cortical merging of sensory inputs in the forepaw area of S1, we used LFPs, single units, and VSDi responses to two-point stimulation. LFPs, recorded in cortical layer 4, are thought to mostly reflect synaptic activity within this layer (Buzsáki et al., 2012), which is driven mainly by direct thalamo-cortical input. At this stage of processing, the spatial distribution of cortical inputs elicited by two-digit costimulation (for example, D2D4) resembles the sum of the corresponding one-digit stimulation (for example, D2 + D4). However, the response amplitude remained lower than the sum of individual components, as if the channel were already saturated. When the cortical LFP representations of the stimulated digits were close in space, e.g., D2D3 and D2D4, the pattern of activation was unimodal and centered between the effectively stimulated digit representations. Conversely, the response to the costimulation of D2 and D5 had two distinct peaks. At the same laminar level, although most neurons did not display a costimulation effect, approximately one-third showed modulations that were compatible with the building of the cortical merging: units representing the edges of the stimulation tended to decrease their response probability, whereas neurons representing the nonstimulated center increased their response probability. For the D2D4 population response, these modulations biased the spatial information toward D3. When the cortical distance was larger, e.g., for D2D5 stimulation, the individual neuron modulations were not sufficient to lead the classifier to choose the center digits. LFP and single units thus show that cortical merging occurs gradually as a function of cortical distance within S1 layer 4, where the synaptic and firing activity is merged for D2D4 stimulation but not for D2D5, although merging-compatible modifications still occurred in the latter case.

Although merging in layer 4, as assessed with electrophysiological recordings, was found to depend on the distance between the costimulated digits, VSDi revealed that in superficial layers 2/3, the cortical pattern of activity resulted from a clear merging regardless of the costimulated digit representational distance. The emergence of activation as probed with VSDi was unimodal and centered, even for D2D5. Altogether, these results suggest that the merging of simultaneous tactile inputs is constructed downstream, as the information passes through the tactile sensory processing pathway.

In all three types of signals recorded (LFP, single units, and VSDi), costimulation induced a greater amount of activity than that evoked by single stimulation. Considering the fact that a multiple-point simulation elicits a more intense sensation than a single-point stimulation (Gardner and Spencer, 1972a), these results suggest that the intensity of the stimulus may be coded by integrated activity over the whole population, rather than the response of neurons located in the center of the representation of a stimulated zone, as argued by Gardner and Costanzo (1980). This increased number of active neurons, promoting an increased spatial summation, could be the substrate for more efficient and faster detection, as is the case for costimulation observed in humans (Hashimoto et al., 1999).

### Interaction of overlapping inputs in cortical merging

To our knowledge, the only previous studies demonstrating such cortical merging of two-point tactile nonvibrissa stimulation used intrinsic signal optical imaging (ISOi) and functional MRI in squirrel monkeys (Chen et al., 2003, 2007; Friedman et al., 2008). The authors did not find cortical merging for nonadjacent digit costimulation, whereas we demonstrated it using VSDi in superficial layers 2/3 not only for D2D3 and D2D4 but also for D2D5 costimulation. Beyond the fact that we used different animal models, it important to note that the duration of the tactile stimulation in the above studies was substantially longer (4 and 30 s vs. 20 ms in our study). VSDi allows a more direct measure of neuronal activity and is able to deliver shorter-duration stimuli (20 ms), as there was no need to wait for the development of a hemodynamic response. Chen et al. (2003, 2007) mainly observed inhibition between inputs from adjacent digits, leading to a smaller activation area, centered at the interface of the two somatotopic areas devoted to each of the stimulated digits. Cortical merging probably involves more than the inhibition of one digit’s afferents onto another’s. In the present study, LFP responses, as well as the size of activation areas evoked by costimulation, were larger than those evoked by single-digit stimulation. Additionally, a substantial proportion of the recorded units representing the center nonstimulated cortical area showed a facilitation effect and fired more than they did when the digits at the edges of the costimulation pattern were stimulated alone.

We showed that two-point stimulation elicits a subadditive cortical response, i.e. weaker than the addition of the two single-stimulation responses, but still greater than that of a single stimulation. This result is in line with the findings of Gardner and Costanzo (1980), who reported a “broadened and flattened” cortical response to triple-point stimulation straddling the center of a neuron’s receptive field. Therefore, our study extends those findings, as we provide evidence for similar units’ modulation to multiple-point stimulation, but in the absence of a central stimulation and with a stimulation pattern that is largely wider than the center of the neurons’ receptive fields. Based on the available evidence, we propose that the interaction of simultaneous inputs coming from two close skin locations leads to both a partial inhibition of the neurons within the corresponding cortical representations and an enhanced response in the cortical area located between these representations.

Important questions remain regarding the location of this interaction in the somatosensory pathway, the mechanisms underlying the integration of concurrent inputs, and the modification of the inhibition–excitation balance. An ISOi study has established that multiple-whisker stimulation evokes a relatively symmetrical activity profile, with a single central peak emerging in the cortex and not upstream (Chen-Bee et al., 2012). Those authors also showed that the spatial distribution of this merged activation can be predicted with a sublinear summation of single whisker–dependent activity. In line with previous studies (Simons, 1983; Mirabella et al., 2001), this suggests that in the cortical superficial layers, a superposition of excitation and reciprocal inhibition sustains the merging of inputs, similar to the addition of so-called Mexican-hat activations, as first proposed by von Békésy (1959).

Our present LFP results corroborate this hypothesis, as the costimulation-evoked activity is well correlated with the sum of activity resulting from the single-digit stimulation. Our KNN decoding procedure of the single-unit responses also indicated that the population activity generated by costimulation resembled the sum of the population responses to the digits alone. Moreover, the observation that the response latency to D2D4 stimulation of neurons representing the central digit D3 was equal to that of the edge neurons corroborates the idea that these central neuronal responses depend on the superposition of the same direct inputs as those received by edge neurons. Indeed, it has been shown that the latencies of S1 neuronal responses to the stimulation of different cutaneous locations are dependent on the distance of the stimulus location, with respect to the receptive field center (Armstrong-James et al., 1991; Moxon et al., 2008). This difference in latency arises from a weaker synchrony in the inputs (Fox, 2008). Based on our current results, we hypothesize that inputs from edge digits could add up and drive the center neurons above spiking threshold, akin to if their preferred digit had been stimulated. In the barrel cortex, layer 4 neurons’ response to adjacent whiskers mainly relies on thalamocortical inputs (Goldreich et al., 1999; Petersen and Sakmann, 2001; Laaris and Keller, 2002). Although the unit responses are less segregated in the forepaw area than in the barrel field, the short response latency to D2D4 stimulation recorded in our study suggests that D3 neurons are primarily driven by thalamic inputs and not by horizontal connections from neighboring digit representational cortical sectors.

If cortical merging results only from the superposition of inputs and their interactions, one would expect that the divergence and convergence of information across the different network layers would allow the merging of inputs coming from more distant locations. In other words, as the size of single-point-stimulation cortical representation increases, the overlap between inputs increases, thus increasing the probability for merging to occur. The size of the cortical representation of a single whisker drastically increases from layer 4 to layer 2/3 (Ferezou et al., 2006). It has been shown that an individual layer 4 neuron contacts ∼300–400 neurons in layer 2/3 and that ∼300–400 layer 4 spiny neurons innervate single layer-2/3 pyramidal cells (Lübke et al., 2003). Moreover, receptive field sizes expand between layers 4 and 2/3 (Sur et al., 1985). We found that in layer 4, D2D5 stimulation does not evoke a merged response, whereas in the more superficial layers 2/3, D2 and D5 inputs are clearly funneled. This laminar difference could be seen as reflecting an intrinsic property of the cortical network.

### Mapping of cortical function onto its laminar structure

Beyond the superposition of excitatory and inhibitory inputs, the recurrent nature of the cortical network could promote the merging process. There is considerable evidence that recurrent processing occurs in the extraction of relevant sensory stimuli features (Lamme and Roelfsema, 2000; Roelfsema et al., 2002; Sillito et al., 2006). Their representation in the primary sensory areas seems to be the result of the confrontation of feedforward and reentrant activities. Most of the models used to demonstrate the importance of recurrence have not taken into consideration the laminar architecture of the cortex and have not specified the contribution of the different cortical layers to this process (Sporns et al., 1991; Lamme and Roelfsema, 2000; Auksztulewicz et al., 2012).

As cortical merging seems to be reinforced across the cortical layers, we needed a model network that takes cortical layer anatomy and physiology into account. With the LaminART model of sensory cortices, Raizada and Grossberg (2003) posit that the cortex is not just a feedforward filter but is designed to “bind together distributed data into coherent groupings.” In this model, the intralaminar horizontal interactions and the layer 2/3 → 6 → 4 and back to 2/3 interlaminar loop give rise to cooperative and competitive interactions, promoting the selection of the strongest 2/3 grouping (i.e., a group of neurons representing elementary perceptual units, like locations within the body map). The gap between the representations of the stimulated digits could be filled by means of this folded-back pathway (Grossberg and Raizada, 2000). As this model gives layers 2/3 the role of forming/selecting coherent groupings, its architecture could account for the merging that we observe for D2 and D5 in these layers, and not in layer 4.

### What tactile illusions reveal about cortical processing

If we adopt a holistic approach in which sensory systems have to bind together segmented bits of sensory information to create a percept, one can think of the funneling process as a product of the Gestalt rule of grouping. Indeed, two stimulations contacting the skin at the exact same time, that are close in space, are most likely to be due to a single object whose center should be between the two points. In this context, our present data are consistent with the view that the somatosensory network is built so as to process simultaneity and proximity as the most relevant cues to parse the world into perceptual objects. In line with this idea of space and time proximity as cues to delineate objects, numerous other tactile spatiotemporal illusions have been described, suggesting that the timing of stimulation influences the spatial representation in the somatosensory system. The tau effect shows that equal distances between two brief stimuli applied on the forearm can be made perceptually different by manipulating the timing of the two-point stimulation (Benussi, 1913; Gelb, 1914; Helson, 1930; Helson and King, 1931). In the saltation illusion, two consecutive taps on the skin are perceived as being applied closer to each another when the delay between them becomes shorter (Geldard, 1982; Kilgard and Merzenich, 1995). Together with sensory funneling, these illusions show that the somatosensory system uses time to compute space. It is as if temporally close stimulations must be processed as a single object that moves with an expected speed. This expected speed could arise from the natural experience of moving objects. A Bayesian model of tactile perception based on these observations has been proposed and validated psychophysically (Goldreich, 2007; Tong et al., 2016). Including a prior that was an expectation for a low-speed stimulus traveling along the skin, it could reproduce numerous psychophysical data from studies investigating all these illusions. In summary, the system is tuned to bind temporally close stimuli as unified objects and uses inferences to improve its estimation for their location on the skin. We hypothesize that, as for the funneling, the S1 representation of a two-point sequence with a non-null interstimulus delay would undergo a spatial distortion within the somatotopic reference map. The cortical activation evoked by the second stimulus could be spatially displaced toward the representation of the first stimulus, in a delay-dependent manner. Wiemer et al. (2000) proposed a model in which the first stimulus-evoked cortical response spreads spatially and increases the excitability of nearby neurons. This local facilitation then favors a spatial shift of the response emergence when a subsequent delayed thalamic input arrives.

The synchrony-dependent spatial distortion showed in the present work, and in other funneling studies, could shed new light on how topographic maps are constructed functionally from an initial connectivity network. It could also provide new insights into the well-documented experience-dependent remodeling of somatotopic maps, in which synchronously delivered repetitive inputs tend to aggregate the cortical representations of paired whiskers (Diamond et al., 1993) or skin surfaces (Allard et al., 1991; Wang et al., 1995; Byl et al., 1996; Rosselet et al., 2008) and alter somatosensory perception accordingly (Elbert et al., 1998; Byl et al., 2000). Inspired by the data of Wang et al. (1995), the aforementioned cortical model (Wiemer et al., 2000) was built with a timing-based auto-organization algorithm. It created somatotopic maps by transforming average interstimulus intervals into representational distances, arguing that the specific cortical organization could explain the formation of both maps and spatiotemporal illusions.

Further investigations are needed to unravel the cortical merging effect. The laminar differences could be confirmed using a microelectrode array and VSDi, through recording responses to digit costimulation in multiple cortical layers simultaneously. The idea of cortical merging as a default *modus operandi* of sensory inputs could be reinforced by verifying its occurrence using other stimuli locations and distances, as well as other sensory modalities. Similar approaches could be employed to investigate the effect of delay onto the cortical representation of a sequentially presented stimuli, as S1 has been shown previously to be strongly involved in the saltation illusion (Blankenburg et al., 2006).

## Synthesis

Reviewing Editor: Leonard Maler, University of Ottawa Decisions are customarily a result of the Reviewing Editor and the peer reviewers coming together and discussing their recommendations until a consensus is reached. When revisions are invited, a fact-based synthesis statement explaining their decision and outlining what is needed to prepare a revision will be listed below. The following reviewer(s) agreed to reveal their identity: Daniel Goldreich

Editor summary

Both reviewers agree that this Ms advances the field of perceptual merging of stimuli. There is a careful analysis of the data resulting from three different methods. Although earlier papers had already suggested that merging occurs in the supragranular layers of cortex, the higher resolution of the methods in this Ms provide strong proof of this notion.

There are numerous edits of the Ms that will be required to tighten up the presentation; right now it is too 'wordy' and the Discussion is too long.

Below I give edited versions of the detailed comments made by the two reviewers.

Reviewer #1

The paper would benefit greatly, however, and garner more widespread interest from readers, if the authors could more clearly relate their findings to the broader perceptual research on tactile spatiotemporal illusions that occur as a function of inter-stimulus interval, of which merging illusions in response to simultaneous (0 ISI) stimuli represent just one extreme case.

Methods

lines 102-107:

The authors state that the forepaw “was glued on a plate, glabrous side up.” Somewhat more detail about this would be useful. In particular, the natural position of the forepaw is glabrous side down. Presumably, then, the paw had to be rotated to an unnatural posture in order to glue it with glabrous side up? Might this have affected blood flow to the paw or otherwise compromised the preparation?

It would be useful to provide more information regarding the tactile stimulation:

How many microns was the “very small indentation"? If a precise measurement is not possible, even an approximate estimate would be appreciated.

What was the contacting probe's shape and surface area?

What was the shape of the driving waveform? (square wave pulse, sinusoidal pulse, ramp-and-hold, etc?).

Discussion

A brief discussion of these additional items would enhance the paper:

1) The authors observe some merging in the layer IV unit activity, albeit limited to about a third of neurons. It could be interesting to speculate briefly on the neural substrate for this merging in layer IV, in light of previous research that has indicated that layer IV whisker barrels are functionally independent - e.g.:

- Laaris and Keller (2002) Functional Independence of Layer IV Barrels. J Neurophysiol 87:1028-1034

- Petersen and Sakmann (2001) Functionally Independent Columns of Rat Somatosensory Barrel Cortex Revealed with Voltage-Sensitive Dye Imaging. J Neurosci 21:8435-8446

- Goldreich, Kyriazi and Simons (1999) Functional Independence of Layer IV Barrels in Rodent Somatosensory Cortex. J Neurophysiol 82:1311-1316.

2) The authors used a fixed very small stimulus amplitude. Could they speculate as to how merging might change as a function of stimulus amplitude? As the cortical spatial activation profile sharpens with increasing stimulus amplitude (see, e.g., Peterson et al (1998) Optical Imaging and Electrophysiology of Rat Barrel Cortex. I. Responses to Small Single-vibrissa Deflections. Cerebral Cortex 8:173-183), would the authors predict that merging would diminish as stimulus amplitude is increased?

3) The relation between the authors' finding and perceptual illusions is very interesting. The two-point stimuli used in the present study were always simultaneously presented. Could the authors speculate on how they might expect merging in the supragranular layers to change as a function of inter-stimulus interval (ISI)? The authors already relate their findings briefly to sensory saltation, an illusion that is evoked by stimuli at short ISI. It would be very interesting to speculate more specifically about the effect of ISI on unit responses, as the perceptual effects of ISI are well-studied. Specifically, at large ISI, two points are perceived to be spatially well-separated, but as ISI is decreased, the two points are perceived to approach one another, finally merging as ISI is decreased towards zero. Goldreich (2007) derived a “perceptual length contraction formula” that described this effect; the formula furthermore predicted that perceptual length contraction would become more pronounced with the use of weaker stimuli. Tong et al (2016) subsequently confirmed these predictions experimentally: Tong J, Ngo V, Goldreich D (2016) Tactile length contraction as Bayesian inference. J Neurophysiol 116: 369-379. Although the authors used exclusively simultaneous stimuli, could they speculate briefly about how the neural responses might change with ISI and stimulus intensity in order to support perceptual length contraction effects?

Minor comments

The authors frequently use the word “repartition.” Perhaps they mean “distribution"? The dictionary definition of repartition is “to divide or partition something again."

line 110: “a 16 channel tungsten microelectrode arrays” should be “a ... array” (singular).

line 114: “the discrimination of waveforms were performed online” should be “the discrimination ... was performed"

line 121: “within the time 40 ms window” should be “within the 40 ms time window"

line 266: “Unit responses where considered as” should be “Unit responses were considered as"

Figure 1A caption: The authors write that “The yellow dot represents the electrode at 0 mm.” There are many yellow dots. I believe they mean the blue dot?

Figure 1B-E caption: The authors state that these data were “recorded from one electrode.” Could they provide the position (mm) of that electrode?

Figure 5 caption: “the units preferred digit” should be “the unit's preferred digit"

line 309-310 and Figure 5 title: In the text, the authors describe “emulating the somatotopic distribution of the units,” and the Figure 5 title is “Emulated cortical pattern from the population responses.” The word “emulate” means to model, mimic, simulate, etc. The authors' use of the word here is confusing, because it gives the impression that the data plotted in Figure 5 are from computer-simulations rather than experimentally observed.

line 370: with respect to Fig 7A, the authors provide the following correct recognition probabilities: “D2: 60.73%; D3: 70.85%; D4: 60.06%.” The values provided for D3 and D4 match those in the figure, but the value provided for D2 (60.73%) is apparently a typographic error, as it does not match that in the figure (65.73%).

lines 375-376: The authors write that “Consistently, the responses of D4 neurons to their preferred digit were classified as if they were evoked by D2 stimulation.” I found this statement to be somewhat confusing. Could the authors please provide more detail? Why do they think this occurred?

Reviewer #2

Major comments

The recording of local field potential to delineate the cortical region activated by stimulation of single digits is questionable. Indeed, LFPs are not as local as the name suggests. (see Kajikawa and Schroeder (2011) How local is the local field potential? Neuron 72: 847-858). The lateral spread of LFPs through volume conduction may be in the millimeter range. Current source density analysis, or at least differential recording would yield a better localization. The authors did not mention where the reference electrode was located. Therefore I consider the result section on LFP not much informative.

The authors also used multiunit recording to localize the foci of excitation in layer 4. This method is more reliable. Although one third of the units in layer 4 showed modulations that were compatible with the building of the cortical merging, most neurons did not display a co-stimulation effect.

The new and most interesting part of the study is the recording of supragranular layers with voltage sensitive dye. VSDi revealed a clear merging of cortical activity in the superficial layers 2/3 regardless of the costimulated-digit representational distance. This result is in line with prior results obtained with intrinsic imaging (Chen et al., 2003). Yet, the spatial and temporal resolution of the VSDi method is far superior to intrinsic optical imaging.

Minor points

In Figure 3 check the gray lines in E-H; the gray tones of the rasters and plots do not correspond.

The Discussion is overly long. It is more appropriate for a PhD thesis than for a scientific article.

## Author Response

Editor summary

Both reviewers agree that this Ms advances the field of perceptual merging of stimuli. There is a careful analysis of the data resulting from three different methods. Although earlier papers had already suggested that merging occurs in the supragranular layers of cortex, the higher resolution of the methods in this Ms provide strong proof of this notion.

There are numerous edits of the Ms that will be required to tighten up the presentation; right now it is too 'wordy' and the Discussion is too long. Below I give edited versions of the detailed comments made by the two reviewers.

We would like to thank the reviewers for their useful comments. We have tried to find a balance between reducing the size of the discussion as requested by both reviewers and the inquiries for including additional discussion points by reviewer 1. All concerns have been addressed below.

In the Manuscript, both added and removed text appear in red.

Reviewer #1

The paper would benefit greatly, however, and garner more widespread interest from readers, if the authors could more clearly relate their findings to the broader perceptual research on tactile spatiotemporal illusions that occur as a function of inter-stimulus interval, of which merging illusions in response to simultaneous (0 ISI) stimuli represent just one extreme case.

This global comment is dealt with in paragraph 3 of the responses related to the Discussion

Methods

lines 102-107:

The authors state that the forepaw “was glued on a plate, glabrous side up.” Somewhat more detail about this would be useful. In particular, the natural position of the forepaw is glabrous side down. Presumably, then, the paw had to be rotated to an unnatural posture in order to glue it with glabrous side up? Might this have affected blood flow to the paw or otherwise compromised the preparation?

The hairy side of the rat left forepaw was glued on a plate placed in a nearly vertical plane, perpendicularly to the table supporting the animal, and formed a natural angle with the forearm. The tips of shafts of custom-made electromagnetic stimulators were placed on the glabrous side of each of four fingertips with the help of a binocular microscope.

The text has been modified accordingly.

It would be useful to provide more information regarding the tactile stimulation:

How many microns was the “very small indentation”? If a precise measurement is not possible, even an approximate estimate would be appreciated. What was the contacting probe's shape and surface area? What was the shape of the driving waveform? (square wave pulse, sinusoidal pulse, ramp-and-hold, etc?).

The probes contacting the skin had a 1 mmÂ² surface area. Stimulation consisted of a ca. 200-300 Âµm square-wave pulse indentation and lasted 20 ms.

See correction in the text.

Discussion

A brief discussion of these additional items would enhance the paper:

1) The authors observe some merging in the layer IV unit activity, albeit limited to about a third of neurons. It could be interesting to speculate briefly on the neural substrate for this merging in layer IV, in light of previous research that has indicated that layer IV whisker barrels are functionally independent - e.g.:

- Laaris and Keller (2002) Functional Independence of Layer IV Barrels. J Neurophysiol 87:1028-1034

- Petersen and Sakmann (2001) Functionally Independent Columns of Rat Somatosensory Barrel Cortex Revealed with Voltage-Sensitive Dye Imaging. J Neurosci 21:8435-8446

- Goldreich, Kyriazi and Simons (1999) Functional Independence of Layer IV Barrels in Rodent Somatosensory Cortex. J Neurophysiol 82:1311-1316.

Although we were asked to reduce the length of the discussion, we shortly discussed the mentioned point. See text modifications in the discussion

"In the barrel cortex, layer 4 neurons' response to adjacent whiskers mainly relies on thalamocortical inputs (Goldreich et al. 1999, Petersen & Sackman 2001, Larris & Keller 2002). Although the unit responses are less segregated in the forepaw area than in the barrel field, the short response latency to D2D4 stimulation recorded in our study suggests that D3 neurons are primarily driven by thalamic inputs and not by horizontal connections from neighboring digit representational cortical sectors."

2) The authors used a fixed very small stimulus amplitude. Could they speculate as to how merging might change as a function of stimulus amplitude? As the cortical spatial activation profile sharpens with increasing stimulus amplitude (see, e.g., Peterson et al (1998) Optical Imaging and Electrophysiology of Rat Barrel Cortex. I. Responses to Small Single-vibrissa Deflections. Cerebral Cortex 8:173-183), would the authors predict that merging would diminish as stimulus amplitude is increased?

This is a very interesting issue to discuss and we provide a response below. However, we did not to add it to the discussion as we have been recommended to shorten it.

We agree on the suggested prediction that the merging diminishes as stimulus amplitude is increased. Indeed, increasing stimulus amplitude, as shown in Peterson et al., 1998, increases the steepness of the evoked cortical activation. The amplitude of the ratio of the center of the activation and its edges increases with the stimulus magnitude, thus leading to a larger contrast of response. These sharpened activations, while still overlapping, are less ambiguous than the ones evoked by weaker stimuli. As a consequence, two well-defined strong stimulations would not be grouped in layer II/III and considered as two different objects. We can thus speculate that for a given skin distance, there is a stimulus amplitude threshold for which cortical processing does not lead to merging. This threshold is expected to increase as distance decreases.

3) The relation between the authors' finding and perceptual illusions is very interesting. The two-point stimuli used in the present study were always simultaneously presented. Could the authors speculate on how they might expect merging in the supragranular layers to change as a function of inter-stimulus interval (ISI)? The authors already relate their findings briefly to sensory saltation, an illusion that is evoked by stimuli at short ISI. It would be very interesting to speculate more specifically about the effect of ISI on unit responses, as the perceptual effects of ISI are well-studied. Specifically, at large ISI, two points are perceived to be spatially well-separated, but as ISI is decreased, the two points are perceived to approach one another, finally merging as ISI is decreased towards zero. Goldreich (2007) derived a “perceptual length contraction formula” that described this effect; the formula furthermore predicted that perceptual length contraction would become more pronounced with the use of weaker stimuli. Tong et al (2016) subsequently confirmed these predictions experimentally: Tong J, Ngo V, Goldreich D (2016) Tactile length contraction as Bayesian inference. J Neurophysiol 116: 369-379. Although the authors used exclusively simultaneous stimuli, could they speculate briefly about how the neural responses might change with ISI and stimulus intensity in order to support perceptual length contraction effects?

We added a short section in the discussion to address this issue.

"We hypothesize that, as for the funneling, the SI representation of a two-point sequence with a non-null inter-stimulus delay would undergo a spatial distortion within the somatotopic reference map. The cortical activation evoked by the second stimulus could be spatially displaced toward the representation of the first stimulus, in a delay-dependent manner. Wiemer et al. (2000) proposed a model where the first stimulus-evoked cortical response spreads spatially and increases the excitability of nearby neurons. This local facilitation then favors a spatial shift of the response emergence when a subsequent delayed thalamic input arrives". We have addressed this question in a companion study whose findings are compatible with this interpretative model.

Minor comments

The authors frequently use the word “repartition.” Perhaps they mean “distribution”? The dictionary definition of repartition is “to divide or partition something again.” We use the term distribution in the revised version of the paper.

line 110: “a 16 channel tungsten microelectrode arrays” should be “a ... array” (singular). Correction made as indicated.

line 114: “the discrimination of waveforms were performed online” should be “the discrimination ... was performed”. Correction has been made.

line 121: “within the time 40 ms window” should be “within the 40 ms time window”. Correction has been made.

line 266: “Unit responses where considered as” should be “Unit responses were considered as”. Correction has been made.

Figure 1A caption: The authors write that “The yellow dot represents the electrode at 0 mm.” There are many yellow dots. I believe they mean the blue dot? We confirm that the blue dot represents the electrode at 0 mm.

Figure 1B-E caption: The authors state that these data were “recorded from one electrode.” Could they provide the position (mm) of that electrode? Precision provided in text.

Figure 5 caption: “the units preferred digit” should be “the unit's preferred digit” Correction has been made.

line 309-310 and Figure 5 title: In the text, the authors describe “emulating the somatotopic distribution of the units,” and the Figure 5 title is “Emulated cortical pattern from the population responses.” The word “emulate” means to model, mimic, simulate, etc. The authors' use of the word here is confusing, because it gives the impression that the data plotted in Figure 5 are from computer-simulations rather than experimentally observed.

We sorted the units according to their preferred digit in order to recreate their putative somatotopic spatial arrangement, and we visualize what the cortical pattern could be like. We agree that the term 'emulated' can be misleading and we propose the following corrective title: 'Cortical pattern reconstruction from the population responses.'

line 370: with respect to Fig 7A, the authors provide the following correct recognition probabilities: “D2: 60.73%; D3: 70.85%; D4: 60.06%.” The values provided for D3 and D4 match those in the figure, but the value provided for D2 (60.73%) is apparently a typographic error, as it does not match that in the figure (65.73%).

This typographic error has been corrected.

lines 375-376: The authors write that “Consistently, the responses of D4 neurons to their preferred digit were classified as if they were evoked by D2 stimulation.” I found this statement to be somewhat confusing. Could the authors please provide more detail? Why do they think this occurred?

We assume that confusion comes from the term 'classified' which usually describes the output of a classifier. In fact, we chose to label the D4 neurons as if they were D2 neurons, and to flip their responses so that a population response labelled with D2 included D2 AND D4 neurons. This was done to compare how the classifier would "see" the costimulation D2D4 versus the sum of D2 and D4.

In the text, we replaced the term 'classified' with 'labelled'.

Reviewer #2

Major comments

The recording of local field potential to delineate the cortical region activated by stimulation of single digits is questionable. Indeed, LFPs are not as local as the name suggests. (see Kajikawa and Schroeder (2011) How local is the local field potential? Neuron 72: 847-858). The lateral spread of LFPs through volume conduction may be in the millimeter range. Current source density analysis, or at least differential recording would yield a better localization. The authors did not mention where the reference electrode was located. Therefore I consider the result section on LFP not much informative.

We used 16-micro electrode arrays (Alpha Omega Eng. with 18 shafts: 16 electrodes arranged in two raws + one ground + one reference). The reference was very close (250 Âµm) to the recording electrode nÂ° 8 (thus a nearby reference). This important information has been added in the text under the following form.

"A reference and a ground electrodes are located near electrode 1 and 8 respectively, thus reducing the uncertainty about the source location of the recorded LFP signal (Kajikawa and Schroeder, 2011). The configuration of the electrode is such that a distance of 250 Âµm separates 2 neighboring electrodes. The 2 rows of 8 electrodes were also separated by 250 Âµm."

A Current Source Density analysis of our LFP data is not relevant as the distance between the different electrodes of each array is too large.

The authors also used multiunit recording to localize the foci of excitation in layer 4. This method is more reliable. Although one third of the units in layer 4 showed modulations that were compatible with the building of the cortical merging, most neurons did not display a co-stimulation effect.

This comment is similar to that formulated by reviewer 1. Wee shortly discussed a possible mechanisms underlying the merging-compatible modulation of one third of the units.

"As in the barrel cortex layer 4 neurons' ability to respond to adjacent whiskers relies mostly on thalamocortical inputs (Goldreich et al. 1999, Petersen & Sackman 2001, Larris & Keller 2002), the short response latency of D3 neurons to D2D4 stimulation suggests that these neurons are driven by thalamic inputs and not by horizontal connections from neighboring digit representations."

Minor points

In Figure 3 check the gray lines in E-H; the gray tones of the rasters and plots do not correspond.

We apologize for the inversion. We modified the colors in the appropriate way.

The Discussion is overly long. It is more appropriate for a PhD thesis than for a scientific article.

We have reduced the length of the discussion, while we tried to be concise in addressing reviewer 1's suggestions.
